# Content and Dietary Contribution Assessment of Mineral Elements in Dairy from Henan Province of China

**DOI:** 10.3390/foods15010135

**Published:** 2026-01-02

**Authors:** Chuanyou Su, Han Li, Yi Li, Chunyu Feng, Tong Fu, Tianliu Zhang, Tengyun Gao

**Affiliations:** Henan International Joint Laboratory of Nutrition Regulation and Ecological Raising of Domestic Animal, College of Animal Science and Technology, Henan Agricultural University, Zhengzhou 450046, China; suchuanyou2010@126.com (C.S.); 17639847356@163.com (H.L.); liyizz123@163.com (Y.L.); 17796666205@163.com (C.F.); futong@henau.edu.cn (T.F.); dairyfarm@163.com (T.G.)

**Keywords:** dairy, magnesium, copper, zinc, tary contribution, nutritional value

## Abstract

To fill the gap in systematic data on mineral contents and their dietary contributions in four mainstream dairy products (sterilized, pasteurized, fermented, modified milk) from Henan Province, China, this study aimed to characterize the mineral profiles [magnesium (Mg), iron (Fe), zinc (Zn), selenium (Se), copper (Cu)] and clarify the mineral nutritional disparities between domestic and imported sterilized milk. A total of 150 samples were analysed via inductively coupled plasma mass spectrometry (ICP-MS). Results revealed significant mineral content differences across dairy types: fermented milk had significantly lower Mg and Zn than sterilized and pasteurized milk (*p* < 0.05). Imported sterilized milk exhibited higher Mg (160.10 ± 31.88 mg/kg) than domestic counterparts (147.41 ± 32.47 mg/kg, *p* < 0.05). In terms of mineral intake contribution rates (defined as the percentage of mineral intake from dairy products relative to the Recommended Nutrient Intake (RNI), unit: %), the rates are ranked in descending order as follows: Se (11.68–25.32%) > Mg (11.11–20.76%) > Zn (5.88–16.33%) > Cu (0.62–1.81%) > Fe (0.25–1.00%). This study elucidates the mineral profiles of Henan’s dairy products, supporting residents’ dairy choices and optimisation of dairy processing technologies.

## 1. Introduction

Milk and dairy products are globally recognised as “nearly perfect” foods due to their balanced nutrition and easy digestibility. They serve as important dietary sources of calcium, magnesium, high-quality proteins, and various trace elements [1,2,3]. However, with the acceleration of industrialisation and urbanisation, the contents of heavy metals and mineral elements in dairy products have become a research focus in both the nutrition and food safety fields, driven by factors such as atmospheric deposition, feed water soil pollution, and metal migration during processing and packaging [4,5]. Yet systematic investigations focusing on specific regions (Henan Province, China) remain scarce, particularly regarding the comprehensive characterisation of essential trace elements and toxic heavy metals in local dairy products, as well as their dietary contribution to the regional population.

On one hand, essential trace elements such as magnesium (Mg), iron (Fe), zinc (Zn), selenium (Se), and copper (Cu)—as key components of minerals—play irreplaceable physiological roles in infant bone development, adult immune regulation, and elderly cognitive protection [6,7,8]. These beneficial intake levels are clearly defined by authoritative guidelines: The Recommended Nutrient Intake (RNI) for Chinese adults is 330 mg/day for Mg, 12–20 mg/day for Fe, 7.5–12.5 mg/day for Zn, 60 μg/day for Se, and 0.8 mg/day for Cu. For infants (0–36 months), intake requirements vary by stage: 0–6 months: 20 mg/day for Mg, 0.3 mg/day for Fe, 1.5 mg/day for Zn, 15 μg/day for Se, and 0.3 mg/day for Cu; 6–12 months: 60 mg/day for Mg, 1.0 mg/day for Fe, 8.0 mg/day for Zn, 20 μg/day for Se, and 0.6 mg/day for Cu; 12–36 months: 500–700 mg/day for Mg, 4 mg/day for Fe, 9.0 mg/day for Zn, 20 μg/day for Se, and 0.8 mg/day for Cu. The RNI for the elderly (≥65 years) is similar to that of adults [9]. When intake levels fall within these ranges, these minerals can fully exert their physiological benefits. Additionally, minerals exert crucial technological effects in dairy products, for instance, by influencing the stability of casein micelles, rennet coagulation properties, and cheese yield [10]. On the other hand, long-term accumulation of toxic elements such as lead (Pb), cadmium (Cd), and arsenic (As)—even at µg/kg levels—may induce kidney damage, neurotoxicity, and carcinogenic risks [11,12].

As China’s third most populous province, Henan had a permanent population of 99.3655 million in 2020, accounting for 7.04% of the national total. Boasting a large population base, it occupies a pivotal position in China’s demographic structure and consumer market [13]. The characteristics of dairy product consumption and mineral dietary intake among residents exhibit significant regional representativeness. Therefore, conducting research targeting this region not only provides a scientific basis for precision nutritional guidance for residents but also offers substantial reference value for national-level dairy nutrition assessment and the formulation of relevant public health policies.

Inductively Coupled Plasma Mass Spectrometry (ICP-MS) has become one of the “gold standards” for multi-element detection in milk, thanks to its ng/L-level detection limit, wide linear range, and isotopic analysis capability [14]. In recent years, scholars at home and abroad have conducted extensive research on single milk types or specific pollution incidents using ICP-MS, demonstrating the method’s universality [15,16]. Furthermore, significant differences exist in the mineral contents of dairy products across different regions. A 2025 study from Pakistan demonstrated that the Fe content in local dairy products reaches as high as 1940 µg/kg, far exceeding that in Germany and Iceland, which further confirms the regional specificity of the mineral profile in dairy products [17]. Milk is defined as a liquid dairy product produced with no less than 80% raw milk (or reconstituted milk) as the primary ingredient, incorporating other raw materials, food additives, or nutritional fortifiers, followed by appropriate sterilization or pasteurization processes [18]. However, there remains a research gap in systematically comparing the mineral element profiles of four mainstream dairy categories (sterilized-pasteurized-fermented-modified) with large samples covering both domestic and imported brands, and further calculating their contribution rates to the Recommended Nutrient Intake for special populations such as pregnant women, lactating mothers, and the elderly.

Based on this, this study collected a total of 150 commercial dairy product samples from 17 cities in Henan Province, comprising 61 samples of sterilized milk, 29 of pasteurized milk, 38 of fermented milk, and 22 of modified milk. Microwave digestion-ICP-MS was used to determine the contents of Mg, Fe, Zn, Se, and Cu. This study evaluated the nutritional contribution rates of these elements to key populations (18–49-year-olds, individuals aged ≥50 years, pregnant women, and lactating mothers) and compared their mineral profiles with international data. This study aims to clarify the quantitative impacts of processing methods and brand origins on the nutritional value of dairy minerals, providing a scientific basis for precise dairy nutrition and standard revision.

## 2. Materials and Methods

### 2.1. Sampling

A total of 150 cow milk-based dairy products from Henan Province were collected in 2023–2024, including 121 domestic products (produced in China) and 29 imported sterilized milk (produced overseas and sold in Henan’s market). This sample size was determined based on previous similar studies on dairy mineral analysis, ensuring sufficient statistical power to detect differences among product types and origins [19,20,21]. The samples consisted of four kinds of dairy products, namely sterilized milk (61), pasteurized milk (29), fermented milk (38), and modified milk (22). Domestic brands refer to dairy products produced in China, while imported brands refer to sterilized milk produced overseas. All samples were collected from commercial markets in 17 cities of Henan Province.

### 2.2. Sample Analysis

To verify the accuracy of the detection method, a national reference material (milk powder CRM, Code: GBW10241, National Institute of Metrology, Beijing, China) was analysed. The recovery rate of the five mineral elements (Mg, Fe, Zn, Se, Cu) ranged from 97.4% to 102.1%, confirming the reliability of the method [22]. The specific steps are as follows: 1.0 g of sample was added to a polyfluoroalkoxy digestion vessel and placed into a microwave digestion tube. Then, 5 mL of nitric acid (65%, Suprapur, Merck, Darmstadt, Germany) and 2 mL of hydrogen peroxide (30%, Suprapur, Merck, Darmstadt, Germany). After overnight predigestion, proceed with digestion according to the specified digestion procedure [23]. The digest was diluted to 25 mL with deionised water and analysed by inductively coupled plasma mass spectrometry (Agilent 7700 Series ICP-MS, Agilent Technologies, Santa Clara, CA, USA) after filtration through a 0.22-µm membrane.

Standard five-point calibrations were developed for each of the mineral elements. The correlation coefficients were >0.9998. To assess the accuracy of the method, milk powder certificate reference material (CRM, Code: GBW10241, National Institute of Metrology, Beijing, China) was analysed. The recovery rates of these five mineral elements from the milk powder certified reference material were 97.35–102.09%.

### 2.3. Contribution Rate Assessment

The exposure of residents to mineral elements from dairy consumption was assessed using the average mineral element content in the test and the recommended milk consumption in China [24], as shown in Equation (1).

EDI = C × DI(1)

where C is the mineral elements content in dairy (mg/kg), DI is the daily milk intake (kg), and EDI is the estimated daily intake (mg/d)

The contribution rate was expressed as CR. It was calculated using Equation (2)

CR = EDI/RNI(2)

where RNI is the recommended nutrient intake (mg/kg/d),based on 310~370 mg Mg, 12~29 mg Fe, 7.5~12.5 mg Zn, 0.06~0.078 mg Se, 0.8~1.4 mg Cu, respectively.

### 2.4. Statistical Analysis

Data analysis was performed using SPSS (IBM, Endicott, NY, USA) version 20. Data were expressed as mean ± standard deviation (SD). Differences in mineral element content among samples were analysed with an independent t-test. *p* < 0.05 was considered statistically significant.

## 3. Results and Discussion

### 3.1. Concentrations of Mineral Elements in Dairy Products

A comprehensive analysis of mineral element concentrations was conducted on a total of 150 dairy product samples collected from the Henan market in China. The study systematically evaluated five essential minerals: Mg, Fe, Zn, Se, and Cu. The samples were categorised into four primary types of dairy products: sterilized milk, pasteurized milk, fermented milk, and modified milk.

Table 1 presents the concentrations of five essential minerals (Mg, Fe, Zn, Se, and Cu) in four categories of dairy products (sterilized milk, n = 61; pasteurized milk, n = 29; fermented milk, n = 38; modified milk, n = 22) from Henan Province, as well as the overall mean value of all 150 samples. Statistical differences between categories are marked with lowercase letters (a, b, c): the same letter indicates no significant difference (*p* > 0.05), while different letters indicate a significant difference (*p* < 0.05). Overall, mineral contents varied significantly by product type, with Mg and Zn showing the most pronounced differences. At the same time, Se and Cu exhibited moderate variations, and Fe had relatively small inter-type differences.

For Mg (mg/kg), the highest concentrations were observed in sterilized and pasteurized milk, with no significant difference; modified milk exhibited an intermediate level, while fermented dairy had the significantly lowest content, approximately 30% lower than that of sterilized milk. In terms of magnesium content (Unit: mg/kg), sterilized milk and pasteurized milk had the highest magnesium contents with no significant difference between them. However, the magnesium content of dairy products in Henan Province not only showed differences among different product types, but also exhibited obvious advantages when compared with the relevant data of dairy products from foreign countries. Available literature reports the magnesium content of Australian dairy products as 101.39 mg/kg [25]. Notably, the magnesium levels of Henan’s dairy products analysed in this study were overall higher than this reference value. This fully demonstrates the excellent performance of Henan dairy products in supplying magnesium nutrients, which can provide local consumers with sufficient magnesium supplementation.

The natural Fe content of milk is extremely low; however, through “nutritional fortification”, it can be transformed into an all-around nutritional drink that provides dual supplementation of calcium and iron. For Fe (μg/kg), the order of average concentrations was sterilized milk > modified milk > pasteurized milk > fermented milk. However, modified milk exhibited a large standard deviation in Fe content, which might be attributed to variations in fortification practices among different brands. For example, Perera et al. [26] investigated iron-fortified milk powder consumed by school-age children (15–16 years old) in Sri Lanka and found significant differences in Fe content among five different brands of modified milk, with a wide concentration range of 6.28–33.72 mg/100 g and an overall large standard deviation (±9.53 mg/100 g).

The average Zn content was 2.45 ± 0.61 mg/kg. After determining the zinc content per kilogram of sterilized milk, pasteurized milk, fermented milk, and modified milk, it was found that the zinc content of fermented milk (1.91 ± 0.48 (b) mg/kg) was significantly lower than that of sterilized milk, pasteurized milk, and modified milk (*p* < 0.05).

The average contents of Se and Cu in the studied milk samples were 30.38 ± 12.79 μg/kg and 28.98 ± 15.18 μg/kg, respectively. Pasteurized milk had the highest Se and Cu contents, which is because it uses low-temperature pasteurization (60–85 °C), which maximises the retention of heat-sensitive Se compounds and reduces their volatilisation [27]. Additionally, the absence of a fermentation process prevents the decrease in soluble Cu content that would be caused by the chelation of Cu by organic acids. Additionally, Chinese market data showed pasteurized milk had significantly higher Se concentrations compared to fermented dairy, attributed to the absence of heat and fermentation-related losses [28]. Table 2 compares the mineral element concentrations of domestic (n = 32) and imported (n = 29) sterilized milk samples, focusing on potential nutritional disparities between the two groups.

In terms of the nutritional value of the four minerals (Fe, Zn, Se, and Cu), domestic sterilized milk is comparable to imported sterilized milk in terms of the nutritional value of Fe, Zn, Se, and Cu; however, it should be noted that consumers with specific magnesium supplementation needs may need to consider other alternatives [29]. This is because there is a significant difference in magnesium content between the two: the magnesium content of imported sterilized milk is (160.10 ± 31.88), which is higher than that of domestic sterilized milk (147.41 ± 32.47, *p* < 0.05).

### 3.2. Contribution Rate Evaluation of Mineral Elements

To evaluate the practical significance of dairy-derived minerals for human mineral nutrition, we calculated the daily mineral intake via dairy consumption (based on the recommended daily intake of 0.3–0.5 kg/d specified in the Dietary Guidelines for Chinese Residents (2022)) and their contribution ratios to the RNIs of different populations (Table 3). The results revealed notable variations in contribution ratios among the five minerals: Se and Mg were the primary contributors, followed by Zn, whereas Fe and Cu exhibited minimal contributions [24].

Table 3 quantifies the intake of these five essential minerals from dairy products, their corresponding RNIs, and the contribution ratios of dairy products to meeting the mineral nutritional requirements of different population groups. All calculations were consistent with the aforementioned dietary guideline, and the table clearly illustrates the logical relationship between “mineral intake–population demand–contribution ratio”.

Among these minerals, Mg intake via dairy consumption ranged from 41.11 to 68.52 mg/d. However, its contribution rate varied slightly due to differences in RNI across populations: the Mg-RNI for adults aged 18–49, the elderly aged ≥50, and lactating women was 330 mg/d, with a corresponding contribution rate of 12.46–20.76%; pregnant women, however, had a higher demand for Mg during pregnancy (RNI = 370 mg/d), leading to a slightly lower contribution rate of 11.11–18.52%. This indicates that dairy products are an important supplementary source of Mg for all population groups. Fe exhibited a low contribution characteristic, with the daily Fe intake from dairy products consistently ranging from 72.23 to 120.39 μg across all populations. Nevertheless, due to the generally high Fe-RNI across different groups (12,000–20,000 μg/d for adults aged 18–49, 12,000 μg/d for the elderly aged ≥50, 20,000–29,000 μg/d for pregnant women, and 24,000 μg/d for lactating women), the contribution rate of dairy products to Fe intake was only 0.25–1.00%, the lowest among the five minerals. This shows that dairy products alone cannot meet the human body’s nutritional needs for Fe. The contribution of Zn was at a moderate level: the daily intake was stable at 0.74–1.23 mg, and differences in contribution rates across populations mainly stemmed from slight adjustments in RNI. The Zn-RNI for adults aged 18–49 and the elderly aged ≥50 was 7.5–12.5 mg/d, with a contribution rate of 5.88–16.33%; the Zn-RNI for pregnant women increased to 9.5 mg/d, resulting in a contribution rate of 7.74–12.89%; and the Zn-RNI for lactating women further rose to 12 mg/d, leading to a contribution rate of 6.13–10.21%. This indicates that dairy products can serve as a regular source of Zn supplementation, but they need to be paired with other foods to meet the body’s requirements. Se was the mineral with the “highest contribution” in dairy products: the daily intake was 9.114–15.19 μg, and the corresponding contribution rate for different populations ranged from 11.68% to 25.32%. The Se-RNI for adults aged 18–49 and the elderly aged ≥50 was 60 μg/d, with a contribution rate of 15.19–25.32%; the Se-RNI for pregnant women was 65 μg/d, resulting in a contribution rate of 14.02–23.37%; and lactating women had the highest Se-RNI (78 μg/d), leading to a contribution rate of 11.68–19.47%. This highlights the core role of dairy products in Se nutritional supplementation. Cu, similar to Fe, made a low contribution to the diet, with a daily intake of 8.694–14.49 μg. The difference in contribution rates was mainly affected by the high RNI of lactating women: the Cu-RNI for adults aged 18–49, the elderly aged ≥50, and pregnant women was 800 μg/d, with a contribution rate of 1.09–1.81%; in contrast, the Cu-RNI for lactating women increased significantly to 1400 μg/d, which reduced the contribution rate to 0.62–1.04%. This indicates that the supplementary effect of dairy products on Cu is limited, and reliance on other dietary sources is necessary.

In general, across all evaluated populations, dairy products are an important source of Mg and Se. Notably, there are two population-specific characteristics: pregnant women had the lowest Mg contribution rate due to elevated Mg RNI, and lactating women had the lowest Cu contribution rate due to higher Cu RNI. These results suggest that dairy products can be a suitable choice for supplementing Mg and Se in daily diets. At the same time, Fe supplementation needs to rely on other food sources (red meat for Fe supplementation and nuts for Cu supplementation) [30,31]. This provides a scientific basis for formulating targeted dietary plans for different population groups and optimising the selection of dairy products.

It should be clarified that among the 150 dairy samples in this study, 121 are domestic products produced in China (including sterilized milk, pasteurized milk, fermented milk, and modified milk), and 29 are imported sterilized milk produced overseas and sold in Henan’s market. All samples were collected from commercial markets in 12 cities of Henan Province. The mineral elements content reported in other studies is shown in Table 4. The Mg content was 137.04 mg/kg, which was comparable to the content range reported in Iceland and higher than the reported values in Brazil and Germany. The Fe content was 240.78 μg/kg, which was higher than the reported value in Germany but significantly lower than those reported in Pakistan and Iceland. The Zn content was 2.45 mg/kg, which was close to the reported value in Pakistan, yet lower than the reported values in Germany and Iceland. The Se content was 30.38 μg/kg; only the Se content in Iceland had been reported, and the result of this study was slightly higher than that range. In contrast, no Se content data was reported in Pakistan, Brazil, or Germany. The Cu content was 28.98 μg/kg, which was higher than the reported value in Brazil but lower than that reported in Germany and Iceland. The differences in elemental content in dairy products can be attributed to the combined effect of multiple factors. First, the comprehensive influence of the natural environment and dairy cow breeds serves as the core foundation. Among these, dairy cow breeds, as a source factor, directly determine the initial level of elemental content in dairy products [32,33]. Furthermore, the feeding and management methods employed during the breeding process, as well as the selection of processing technologies in subsequent production stages, will further amplify this difference in elemental content, ultimately leading to significant variations in the elemental composition of different dairy products [34,35].

## 4. Conclusions

This study systematically analyzed the content characteristics of five essential minerals (Mg, Fe, Zn, Se, and Cu) in commercially available dairy products from Henan Province, China. The results indicated that mineral contents varied significantly by product type: fermented milk exhibited notably lower levels of Mg and Zn, while imported sterilized milk had significantly higher Mg content compared to domestic counterparts. Dietary contribution assessment revealed that daily consumption of 300–500 g of dairy products could provide 11–22% of the RNI for Mg and 12–25% for Se, making dairy products an important source of these two nutrients. The contribution to Zn intake was moderate, whereas contributions to Fe and Cu intake were negligible. Compared with international data, dairy products from Henan Province exhibited certain competitiveness in terms of Mg and Se contents. These findings provide a scientific basis for residents to make rational choices of dairy products, for the dairy industry to optimize production processes (particularly to reduce nutrient loss during fermentation), and for the revision of relevant nutritional standards. Future research could expand the range of elements investigated and further explore the effects of processing parameters on mineral stability.

## Figures and Tables

**Table 1:** Comparison of mineral elements among various of dairy products (mean ± SD).

Mineral Element	Unit	Sterilized Milk (n = 61)	Pasteurized Milk (n = 29)	Fermented Milk (n = 38)	Modified Milk (n = 22)	Total (n = 150)
Mg	mg/kg	153.44 ± 32.56 ^a^	144.09 ± 32.19 ^a^	107.78 ± 33.83 ^c^	132.77 ± 33.17 ^ab^	137.04 ± 37.56
Fe	μg/kg	260.07 ± 97.35 ^a^	228.57 ± 57.88 ^ab^	202.44 ± 77.63 ^b^	269.62 ± 162.51 ^ab^	240.78 ± 102.57
Zn	mg/kg	2.64 ± 0.56 ^a^	2.55 ± 0.37 ^a^	1.91 ± 0.48 ^b^	2.72 ± 0.67 ^a^	2.45 ± 0.61
Se	μg/kg	28.21 ± 10.27 ^b^	40.09 ± 17.17 ^a^	26.77 ± 10.74 ^b^	29.85 ± 9.58 ^ab^	30.38 ± 12.79
Cu	μg/kg	32.38 ± 9.61 ^a^	34.78 ± 26.62 ^a^	20.44 ± 9.35 ^b^	26.68 ± 7.24 ^ab^	28.98 ± 15.18

Note: Lowercase letters (a, b, c) in the same row indicate significant differences among dairy product types (independent *t*-test, *p* < 0.05). Values with the same letter are not significantly different. SE = standard error; n = sample size.

**Table 2:** Mineral elements content in domestic and imported sterilized milk (mean ± SD).

Mineral Element	Unit	Domestic Sterilized Milk (n = 32)	Imported Sterilized Milk (n = 29)
Mg	mg/kg	147.41 ± 32.47 ^b^	160.10 ± 31.88 ^a^
Fe	μg/kg	260.82 ± 96.20	259.25 ± 100.30
Zn	mg/kg	2.72 ± 0.64	2.53 ± 0.45
Se	μg/kg	29.41 ± 9.19	26.90 ± 11.35
Cu	μg/kg	32.57 ± 9.89	32.20 ± 9.93

Note: Lowercase letters (a, b) in the same row indicate significant differences between domestic and imported sterilized milk (independent *t*-test, *p* < 0.05). Values with the same letter are not significantly different. SD = standard deviation; n = sample size.

**Table 3:** Mineral intake, RNI, and contribution rates from dairy products by population group.

Mineral Element	Average Content in Dairy Products	Daily Dairy Product Intake	Age/Stage	Daily Mineral Intake from Dairy	RNI of Mineral	Contribution Rate (%)
Mg	137.04 (mg/kg)	0.3–0.5 (kg/d)	18–49	41.11–68.52 (mg/d)	330 (mg/d)	12.46–20.76
			≥50 years old	41.11–68.52 (mg/d)	330 (mg/d)	12.46–20.76
			Pregnancy	41.11–68.52 (mg/d)	370 (mg/d)	11.11–18.52
			Lactation Period	41.11–68.52 (mg/d)	330 (mg/d)	12.46–20.76
Fe	240.78 (μg/kg)	0.3–0.5 (kg/d)	18–49	72.23–120.39 (μg/d)	12,000~20,000 (μg/d)	0.25–1.00
			≥50 years old	72.23–120.39 (μg/d)	12,000 (μg/d)	0.60–1.00
			Pregnancy	72.23–120.39 (μg/d)	20,000~29,000 (μg/d)	0.25–0.60
			Lactation Period	72.23–120.39 (μg/d)	24,000 (μg/d)	0.30–0.50
Zn	2.45 (mg/kg)	0.3–0.5 (kg/d)	18–49	0.74–1.23 (mg/d)	7.5–12.5 (mg/d)	5.88–16.33
			≥50 years old	0.74–1.23 (mg/d)	7.5–12.5 (mg/d)	5.88–16.33
			Pregnancy	0.74–1.23 (mg/d)	9.5 (mg/d)	7.74–12.89
			Lactation Period	0.74–1.23 (mg/d)	12 (mg/d)	6.13–10.21
Se	30.38 (μg/kg)	0.3–0.5 (kg/d)	18–49	9.11–15.19 (μg/d)	60 (μg/d)	15.19–25.32
			≥50 years old	9.11–15.19 (μg/d)	60 (μg/d)	15.19–25.32
			Pregnancy	9.11–15.19 (μg/d)	65 (μg/d)	14.02–23.37
			Lactation Period	9.11–15.19 (μg/d)	78 (μg/d)	11.68–19.47
Cu	28.98 (μg/kg)	0.3–0.5 (kg/d)	18–49	8.69–14.49 (μg/d)	800 (μg/d)	1.09–1.81
			≥50 years old	8.69–14.49 (μg/d)	800 (μg/d)	1.09–1.81
			Pregnancy	8.69–14.49 (μg/d)	800 (μg/d)	0.97–1.61
			Lactation Period	8.69–14.49 (μg/d)	1400 (μg/d)	0.62–1.04

Note: “Average Content in Dairy” refers to the mean concentration of minerals in 150 dairy samples from Henan Province. “Daily Mineral Intake from Dairy” is calculated using the formula: EDI = C × DI, where C = average mineral content in dairy (mg/kg or μg/kg), and DI = recommended daily dairy intake (0.3–0.5 kg) specified in the Dietary Guidelines for Chinese Residents (2022). “RNI (Recommended Nutrient Intake)” is cited from the Dietary Guidelines for Chinese Residents (2022) [24]. “Contribution Rate” is calculated using the formula: CR = (EDI/RNI) × 100%, representing the proportion of mineral needs met by daily dairy consumption.

**Table 4:** Mineral elements content in dairy from different countries.

Year	Country	Types of Dairy	Content	Reference
2025	Pakistan	Mg (mg/kg)	−	[17]
		Fe (μg/kg)	1940	
		Zn (mg/kg)	2.3	
		Se (μg/kg)	−	
		Cu (μg/kg)	−	
2018	Brazil	Mg (mg/kg)	72.73	[[36]
		Fe (μg/kg)	−	
		Zn (mg/kg)	−	
		Se (μg/kg)	−	
		Cu (μg/kg)	<13.32	
2019	Germany	Mg (mg/kg)	102	[37]
		Fe (μg/kg)	160	
		Zn (mg/kg)	3.75	
		Se (μg/kg)	−	
		Cu (μg/kg)	70	
2011	Iceland	Mg (mg/kg)	100~150	[38]
		Fe (μg/kg)	300~500	
		Zn (mg/kg)	3~5	
		Se (μg/kg)	15~30	
		Cu (μg/kg)	100~300	
2025	China	Mg (mg/kg)	137.04	This study
		Fe (μg/kg)	240.78	
		Zn (mg/kg)	2.45	
		Se (μg/kg)	30.38	
		Cu (μg/kg)	28.98	

## Data Availability

None of the data were deposited in an official repository. The data that support the study findings and models are available from the authors upon reasonable request.

## Abbreviations

The following abbreviations are used in this manuscript:

Mg	Magnesium
Fe	Iron
Zn	Zinc
Se	Selenium
Cu	Copper
Pb	Lead
Cd	Cadmium
As	Arsenic
ICP-MS	Inductively Coupled Plasma Mass Spectrometry
RNI	Recommended Nutrient Intake
